# The Syndrome of Irreversible Lithium-Effectuated Neurotoxicity: A Report of Two Cases

**DOI:** 10.1192/j.eurpsy.2025.788

**Published:** 2025-08-26

**Authors:** G. Ozmen, V. Kolcu, E. Sipahi, B. Yıldırım Cinek

**Affiliations:** 1 Health Sciences University, Erenkoy Mental Health and Neurological Diseases, Training and Research Hospital, Istanbul, Türkiye

## Abstract

**Introduction:**

Lithium is a widely used treatment for mood disorders, particularly bipolar disorder, but its narrow therapeutic range often leads to toxicity. A major complication is lithium-induced neurotoxicity, which is generally reversible with dose adjustment or discontinuation. However, symptoms persisting beyond two months after cessation are deemed irreversible and may result in permanent neurological damage(Verdoux *et al*. Encephale 1991;17:221-4). The permanent sequelae, first recognized in the 1980s, are known as “Syndrome of Irreversible Lithium-Effectuated Neurotoxicity” (SILENT). SILENT is marked by irreversible neurological damage, including cerebellar dysfunction, dementia, parkinsonian syndromes, choreoathetosis, brainstem syndromes and peripheral neuropathies (Farouji et al. Cureus 2023;15).

**Objectives:**

This case report aims to highlight the rare SILENT syndrome and underscore the importance of early diagnosis and management of lithium-induced neurotoxicity.

**Methods:**

**Case 1:** A 61-year-old male with a long-standing diagnosis of bipolar disorder, managed since age 18, presented in 2022 with speech and gait disturbances while on lithium therapy. His lithium level was elevated (2.31 mmol/L), and he underwent emergency hemodialysis after the suspected interaction of NSAIDs with lithium. Despite normal brain imaging, the patient experienced persistent symptoms of postural instability, ataxic gait, dysarthria and tremor over two years. Subsequent imaging revealed cerebral atrophy and ischemic white matter changes. Neuropsychological testing showed frontal-type memory deficits, leading to a diagnosis of Syndrome of Irreversible Lithium-Effectuated Neurotoxicity.

**Case 2:** A 71-year-old male with a 40-year history of bipolar disorder presented with tremors, bradykinesia, dysarthria and anorexia. Blood tests showed renal impairment (creatinine 2.3 mg/dL) and elevated lithium levels (1.7 mmol/L), likely secondary to chronic kidney disease. Lithium was discontinued, and valproate was initiated. Nine weeks later, he returned with increased energy, insomnia, impulsivity, auditory hallucinations, temporal disorientation, perseverative speech, and gait instability. Examination revealed agitation, a blank stare, mild dysarthria and gait imbalance despite normal routine blood tests.

**Results:**

Lithium poisoning is a common clinical issue. Elevated lithium levels can result from excessive intake, impaired excretion or drug interactions. SILENT syndrome, a rare complication of lithium therapy, leads to permanent neurological damage, including cerebellar dysfunction, ataxia, dysarthria and tremor (Konieczny *et al*. Alpha Psychiatry 2024; Mar.).

**Conclusions:**

This emphasizes the importance of monitoring for drug interactions and conducting regular neurological assessments to detect and manage lithium-related complications early. The case underscores the need for heightened clinical awareness to prevent permanent lithium neurotoxicity.

**Disclosure of Interest:**

None Declared

